# Serological and Molecular Detection of *Bartonella henselae* in Cats and Humans From Egypt: Current Status and Zoonotic Implications

**DOI:** 10.3389/fvets.2022.859104

**Published:** 2022-04-14

**Authors:** Amal S. M. Sayed, Reem M. Alsaadawy, Magda M. Ali, Rawhia F. Abd El-Hamid, Roua Sami Baty, Ehab Kotb Elmahallawy

**Affiliations:** ^1^Department of Zoonoses, Faculty of Veterinary Medicine, Assiut University, Asyut, Egypt; ^2^Department of Surgery, Anesthesiology and Radiology, Faculty of Veterinary Medicine, Assiut University, Asyut, Egypt; ^3^Department of Microbiology and Immunology, Faculty of Medicine, Assiut University, Asyut, Egypt; ^4^Department of Biotechnology, College of Science, Taif University, Taif, Saudi Arabia; ^5^Department of Zoonoses, Faculty of Veterinary Medicine, Sohag University, Sohag, Egypt

**Keywords:** serology, molecular, *Bartonella*, cats, humans, Egypt

## Abstract

Bartonellosis is a vector-borne zoonotic disease caused by the intracellular bacterium of genus *Bartonella*. The disease has a worldwide distribution and cats represent the major reservoir of this disease. Despite its global distribution, very limited previous studies have investigated the occurrence of bartonellosis in cats and their owners in Egypt. In an endeavor to explore this topic, we investigated the occurrence of *Bartonella henselae (B. henselae*) infection in 225 samples (blood, saliva, and claw) obtained from 75 healthy cats in Upper Egypt. These samples were routinely obtained during veterinary clinic visits. This study also involved an examination of 100 humans, including cat owners and people with a history of contact with cats. Attempted isolation and identification of *B. henselae* in cats were also performed. Furthermore, PCR was performed for molecular identification of *B. henselae* in blood samples from cats. Meanwhile, an immunofluorescent assay was performed to study the seroprevalence of *B. henselae* infection in humans. In this study, *B. henselae* could not be isolated from any of the examined blood, saliva, or claw samples from cats. Interestingly, *B. henselae* was identified molecularly in 8% (6/75) of blood samples from cats. The seroprevalence of *B. henselae* in humans was 46% and its occurrence was higher in females (46.6%) than in males (41.7%) (*P* = 0.748). *B. henselae* infection was higher among cat owners [51.4% (19/37)] than among people with a history of contact with cats [42.9% (27/63)] (*P* = 0.410). Infection was higher in rural regions [79.5% (31/39)] than in urban regions [24.6% (15/61)] (*P* < 0.001). Collectively, this data provide interesting baseline information about the occurrence of *B. henselae* in cats and humans in Upper Egypt, which reflects the potential zoonotic transmission of this bacterium. Future study is mandatory to explore the occurrence of *B. henselae* in major reservoirs in Egypt.

## Introduction

Bartonellosis is a vector-borne zoonotic disease with a worldwide distribution (1–3). This disease is mainly caused by the Gram-negative facultative intracellular bacterium of genus *Bartonella* (4). Among others, infection by *Bartonella henselae* (*B. henselae*) is considered the most common species of the bacterium, which is associated with cat-scratch disease (4, 5). The epidemiological profile of this disease includes humans and a wide range of mammalian hosts, mainly companion animals. Cats, particularly kittens, represent the major reservoir of *B. henselae* (6). Furthermore, a wide range of blood-sucking arthropods, such as fleas, biting flies, sandflies, mosquitos, lice, and ticks, have been considered as competent vectors. In addition, some studies have revealed that rodent-associated *Bartonella* species and their ectoparasites in different regions of the world can cause bartonellosis in humans (7, 8). Exposure to infected flea feces appears to be the main route of infection in cats, while some other reports have indicated that ingestion of infected fleas or infected feces could facilitate transmission (9, 10). Another previous study (11) illustrated that ticks (mainly *Ixodes ricinus)* may play a role in the transmission of *B. henselae* and other *Bartonella* species among cats through trans-stadial transmission. Humans can contract bartonellosis through bites or scratches from cats, which act as mechanical methods of transmission, in addition to skin lesions infected by inanimate objects, such as thorns or pins (12).

In accordance with its worldwide distribution and presence as a public health concern, the etiological agent is distributed on all the continents among major reservoirs. For example, ~12,000 Americans contract bartonellosis each year and around 500 of these are admitted to hospitals (13). In accordance with its clinical impact, *B. henselae* has been implicated as a cause of acute bacterial disease, which primarily affects the lymph nodes, skin, and internal organs. The reported series of symptoms includes fever, erythematous papules, pustules, ulcers, and unilateral lymphadenitis (14). Naturally infected cats are mostly asymptomatic, but some animals develop cardiac syndromes, such as endocarditis or myocarditis, in addition to ocular complications in some cases (15). Although the prevalence of *B. henselae* infection in cats significantly fluctuates, the highest rates of infection occur in temperate regions where conditions are most favorable for the development of *Ctenocephalides felis* (16). Meanwhile, in humans, *B. henselae* can result in a series of diseases, such as lymphadenopathy, bacteremia, bacillary angiomatosis, and bacillary peliosis (17). Revising the available literature, very limited previous studies have investigated the occurrence of *B. henselae* in Egypt. Thus, this study aimed to explore the potential role of cats as a major reservoir of *B. henselae* infection. Moreover, this study explored the seroprevalence of *B. henselae* infection in humans who come into contact with infected animals.

## Materials and Methods

### Ethical Considerations

This study was ethically reviewed and approved by the Scientific Research Committee and Ethics Board of Assiut University, Egypt (institutional review board ethics approval number: 17300307).

### Sampling

#### Cat Samples

A total of 225 samples were collected from 75 apparently healthy cats from Asyut Governorate, Egypt, during the period from March 2016 to March 2018. These samples were routinely collected during veterinary clinic visits. The full details of the study cohort of cats are shown in Table 1. Three types of sample, namely, blood, saliva, and claw, were collected from each cat. For blood samples, 1 ml of blood was collected from the cephalic vein of each cat under complete aseptic conditions in sodium citrate vacuum tubes before being stored at −20°C for culture examination. Salivary and oral swabs were taken using a sterile cotton applicator placed against the inside surface of each cat's cheek. These swabs were then suspended in 5 ml of brain heart infusion (BHI) broth with *Brucella* growth supplement, as previously described (18). Meanwhile, claw samples were collected in 5 ml of BHI broth (Biolife, code: 1230) with *Brucella* growth supplement (18).

**Table 1:** The full details of the study cohort for each of the enrolled cat's sex, age, and living habits.

		**Enrolled cats**
Sex	M	33
	F	42
Age (months)	3–6	15
	7–24	54
	25–48	6
Living habits	Household cats	31
	Stray cats	5
	Pet shop cats	39

#### Human Samples

A total of 100 blood samples were collected from human participants. Specifically, 37 samples were collected from owners of cats (*N* = 37) and 63 samples were collected from people with a history of contact with cats (*N* = 63) that means those people had a history who raised cats in their homes. All the participants were healthy and the full details of the study cohort and their results are shown in Table 2. Blood samples were collected without anticoagulation from humans and collection tubes were left in a standing position for 20–30 min before being centrifuged at 3,000 rpm for 15 min. Sera samples were stored at −20°C until further examination.

**Table 2:** Seroprevalence and epidemiological data associated with the occurrence of Bartonella henselae (*B. henselae*) infection in humans.

**Demographic data**	**No. of samples**	***B. henselae*** **positive samples**		***P*** **-value**
		**Number**	**%**	
**Sex**
Male	12	5	41.7	*P* = 0.748
Female	88	41	46.6	
Total	100	46	46	
**Age (years)**
10–20	8	1	12.5	*P < * 0.001
21–30	55	33	60	
31–40	27	12	44.4	
More than 40	10	0	0	
Total	100	46		
**Residence**
Urban	61	15	24.6	*P < * 0.001
Rural	39	31	79.5	
Total	100	46		
**Contact with cats**
Cat owner	37	19	51.4	*P* = 0.410
Previous history of contact with cat	63	27	42.9	
Total	100	46		

### Isolation of *Bartonella henselae* From Cat Samples

Blood samples from cats were defrosted and centrifuged at 3,800 rpm for 70 min, as described previously (19). The pellets were then inoculated on BHI agar (Biolife, Ref. 4012352) containing 5% sheep blood that was confirmed to be *Bartonella* negative (20, 21) and 2% *Brucella* growth supplement (HiMedia, FD005) and then incubated at 35°C in an atmosphere of 5% carbon dioxide (CO_2_) for 4–8 weeks (18, 22). Oral swabs and claw samples were enriched in 5 ml of BHI broth containing 5% sheep blood and *Brucella* growth supplement, before being incubated at 35°C in an atmosphere of 5% CO_2_ for 10 days (21, 23, 24). A loopful of each sample was plated on BHI agar with 5% sheep blood and 2% *Brucella* growth supplement, before being incubated at 35°C in an atmosphere of 5% CO_2_ for 4–8 weeks (18, 22). The cultured plates were continuously checked following inoculation for any colony formation. If any, the pure colonies were then subjected to identification through colony morphology, Gram staining, and certain biochemical tests, including the oxidase and catalase tests (25).

### Molecular Detection of *Bartonella henselae* Among Cats

This step involved molecular detection of *B. henselae* in cat samples, which was performed as described below.

#### Deoxyribonucleic Acid Extraction From Blood Samples

Deoxyribonucleic acid extraction from the blood samples of cats was performed using the QIAamp DNA Mini Kit (Ref. 51403) according to the manufacturer's instructions.

#### Polymerase Chain Reaction Amplification

This step involved targeted amplification of the citrate synthase gene *(gltA)* of *B. henselae*, as described previously (26) with slight modifications. The following species-specific primers were used: BartogltA forward: 5′-TTCCGYCTTATGGGTTTTGG-3′ and Bartohenselae: 5′-CATTTCTGTTGGAAATCCTAG-3' with an amplicon size of 246 bp. A thermal cycler (Biometria, T Professional) was used for DNA amplification. Briefly, the PCR reaction mixture volume was 25 μl, which comprised 13 μl of master mix, 1 μl of forward primer (0.40 p/mol), 1 μl of reverse primer (0.40 p/mol), and 100 pg/μl to 100 ng/μl DNA template and deionized distilled water was added to the reaction to make up the final volume. The amplification included an initial denaturation step of 5 min at 95°C, followed by 35 cycles of 45 s at 95°C, 45 s at 56°C, and 45 s at 72°C, with a final extension step at 72°C for 7 min. Five microliter of each of the resultant amplified PCR products was analyzed in 1.6% (w/v) agarose gels in Tris-acetate-ethylenediaminetetraacetic acid (EDTA) buffer stained with ethidium bromide, transilluminated under ultraviolet light, and photographed. The AMPLIRUN® *B. henselae* DNA Control (Vircell, Ref MBC005) was used as the positive control, while purified water was used as the negative control. The controls were processed in parallel with the experimental samples to detect possible contamination.

### Serological Detection of *Bartonella henselae* in Human Sera Samples

In this step, an indirect immunofluorescence immunoglobulin G (IgG) kit of *B. henselae* (Vircell, lot 16 BHQ307) was used to examine human sera samples according to the manufacturer's instructions. A *B. henselae* IgG antibody titer of ≥1:64 was considered as an indicator of infection.

### Statistical Analysis

All the data were analyzed using Statistical Package for the Social Sciences (SPSS) software version 17. The data were subjected to ANOVA using the chi-squared procedure of SPSS software. A probability value (*P*-value) of < 0.05 was considered as statistically significant.

## Results and Discussion

### Detection of *Bartonella* spp. in Samples From Cats

The diagnosis of zoonotic pathogens in major reservoirs remains one of the main methods to control this category of diseases (27–31). This study provides a novel contribution by investigating the occurrence of *B. henselae* in cats and humans in Egypt. Attempts were also made to isolate and identify *B. henselae* from cats bacteriologically. *B. henselae* was identified in cats using molecular methods. Serological detection of *B. henselae* IgG from human sera samples was also achieved using indirect immunofluorescence.

In this study, *B. henselae* could not be isolated on BHI agar from any of the examined blood, saliva, or claw samples from cats, which is consistent with several previous studies (18, 32). The inability to culture *B. henselae* may be due to the difficulty in isolating *B. henselae* or due to the presence of inactive bacteria (non-culturable) in the examined samples, as reported in a previous study (33). However, it should be taken into consideration the incubation time or the medium used and the level of bacteremia that might influence the isolation of *Bartonella* on culture, in addition to the fastidious nature of this bacteria, making it problematic to isolate from culture media and lower the sensitivity of this method (23, 34–36). In contrast, *Bartonella* spp. were previously cultured from blood samples obtained from 7% (7/100) of cats in Spain (37). It is noteworthy that molecular methods remain among the most accurate for the detection of pathogens in their major reservoirs (38). In accordance with the molecular detection of *B. henselae* in blood samples from cats (Table 3), a total of 75 blood samples from cats were examined. The results showed that 8% (6/75) of the samples were positive for *B. henselae*. Similar results were reported in cats from the Czech Republic (8%) (39), Turkey (8.2%) (40), and Switzerland (8.3%) (41). However, lower prevalences of *B. henselae* were reported in previous studies in different countries, including Portugal (6.7%) (42) and Sweden (2.2%) (43), using PCR. In stark contrast, a previous serological study in Egypt reported a prevalence rate of 59.6 in cats from Cairo, Egypt (44). Likewise, higher rates of *B. henselae* infection were detected in Japan (9.1%) (45), the United Kingdom (9.4%) (46), Germany (13%) (22), South Brazil (17.02%) (47), Italy (18%) (48), South Korea (33.3%) (49), and Poland (40.48%) (50). The differences in the reported rates of infection among cats in different countries might be attributed to the influence of climatic conditions because the gradient of infection increases from cold climates (0% in Norway) to warm and humid climates (68% in the Philippines) (51). However, the influence of flea infestation, which is more likely in warm and humid areas than in cold areas, should also be considered (52, 53). Furthermore, other factors, including sample size, environmental conditions, hygienic practices and socioeconomic level, type of diagnostic method (either serological or molecular), type of PCR and primers used, and the studied population (stray vs. household cats), might influence the differences in the reported rates of infection by *B. henselae* among cats in different countries (35, 54–58). It is evident that the molecular detection of *B. henselae* is more reliable and sensitive than culture techniques to identify the microorganism, as documented in several previous studies (47, 59, 60).

**Table 3:** Correlation between the results *B. henselae* infected cats and their corresponding household.

**Serial No. and living habits**	**Infected cats**		**Cat household**		
	**Age (months)**	**Sex**	**Age (years)**	**Sex**	***B. henselae*** **IgG antibodies**
1 (Household cats)	24	Male	15	Female	+
2 (Household cats)	18	Male	47	Male	–
			44	Female	–
3 (Household cats)	8	Female	18	Female	–
			52	Female	–
4 (Stray Cat)	6	Female	Not included in the study (Cats were stray or retrieved from pet shops)		
5 (Pet shop)	12	Female			
6 (Pet shop)	12	Male			

### Seroprevalence of *B. henselae* Infection in Humans

Several serological techniques have been developed as cheap and rapid methods to diagnose many infectious agents. Given the fact that it is difficult to isolate *Bartonella* spp. from major reservoirs, serological tests (mainly immunofluorescent assay) confer many advantages in the diagnosis of bartonellosis in humans (61). In this study (Table 2), the overall seroprevalence of *B. henselae* IgG in human sera samples was 46%, which is higher than that reported in a previous study in China (9.68%) (62). However, the seroprevalence in this study was lower than that recorded in veterinarians and cat owners in Poland (53.3%) (63). This variation in the reported seroprevalence might be associated with the risk of *Bartonella* infection in humans, which is lower at northern latitudes than in countries with warm climates (6).

In accordance with the studied epidemiological pattern and potential risk factors for infection, which are shown in Table 2, this study revealed that the rate of infection in females (46.6%) was higher than in males (41.7%), although this difference was not statistically significant (*P* = 0.748). Similar results were reported in a previous study in the United States (13). However, no significant difference in the seroprevalence of *B. henselae* between males (7%) and females (4.7%) was reported in Thailand, which is in harmony with our present findings (54). This difference in the prevalence of *B. henselae* between males and females in various studies might reflect that sex is not a risk factor for *B. henselae* infection. Importantly, as shown in Table 2, the highest rate of *B. henselae* infection in humans (60%) was observed in the 21–30-year age group, followed by the 31–40-year age group (44.4%), while the lowest seroprevalence (12.5%) was detected in the 10–20-year age group. None of the groups aged >40 years demonstrated *B. henselae* infection, but the differences between the age groups were significant (*P* < 0.0*01*). Similar results were reported in a previous study by Maruyama et al. (54) conducted in Thailand. On the contrary, no difference in IgG seropositivity was found between children and adults in another study in Croatia (64). Several previous studies revealed that bartonellosis occurs more frequently among children (65–67). The differences in prevalence rates and its association with age as a potential individual variable factor could be attributed to the development of immunity with age, specific host–pathogen interactions, and history of previous exposure to infection, which results in an acquired immunity following exposure (64, 68, 69).

In terms of the seroprevalence of *B. henselae* infection in humans in relation to residence (Table 2), a significantly higher rate (*P* < 0.001) of *B. henselae* infection was observed in humans in rural (79.5%) compared with urban (24.6%) areas. The present results are in harmony with those reported in another study (70). In stark contrast, no significant difference in IgG positivity was recorded among Croatian patients living in urban areas (44.1%) and others living in rural areas (44.8%) (64). The possible explanation for this high seroprevalence of *B. henselae* in rural areas could be the lower socioeconomic level and poorer hygienic measures in these regions, lack of awareness on dealing with cat scratches and bites, and a higher incidence of flea infestation in these regions than in urban areas (71). Table 2 depicts that the seroprevalence of *B. henselae* in cat owners was higher (51.4%) than in people with a history of contact with cats (42.9%), although the difference was not significant (*P* = 0.410). The high seroprevalence of *B. henselae* reported among cat owners was expected and it is related to continuous exposure to the same source of infection because cat owners are at a high risk of scratches and bites. Along the same line, the high rate of seropositivity for *B. henselae* was estimated in cat owners in Korea (72). The data of the correlation between the infected cats and their households are shown in Table 3. As depicted in this Table, six cats were found infected with *B. henselae* and their age ranged from 6 to 24 months. In accordance with households, only three households of those corresponding infected cats were included in this study. Meanwhile, the households of the remaining three positive cats were not included, since these three positive cats were either stray or retrieved from pet shops. It is noteworthy to state that among other positive cats, there was one cat (cat number 1) and its owner was found infected with *B. henselae*. On the other hand, the remaining households did not carry antibodies against *B. henselae*. Although households in these latter cases were serologically negative, they remain exposed to infection from their infected cats. Collectively, the present serological data provide novel contributions about the high occurrence of *B. henselae* among humans in Egypt.

## Conclusion

This study reports that the occurrence of *B. henselae* infection is high among cats and high exposure of cat owners and people with a history of exposure to cats to the pathogen, reflecting the potential zoonotic transmission cycle between cats and humans. Clearly, this data highlight the benefits of the use of serological and molecular methods in the diagnosis of *B. henselae* infection in major reservoirs. The present findings also prompt local health authorities to take measures to increase the awareness of the public and immunocompromised individuals of the role of cats in transmitting bartonellosis and the importance of keeping cats indoors and controlling flea infestation. Future study is recommended to explore the occurrence and epidemiological pattern of *B. henselae* in Egypt on a large scale, followed by genetic characterization of the circulating species in the country.

## Data Availability Statement

The original contributions presented in the study are included in the article/supplementary material, further inquiries can be directed to the corresponding author/s.

## Ethics Statement

The study was ethically reviewed and approved by the Scientific Research Committee and Ethics Board of Assiut University, Egypt. The ethical approval number is IRB No: 17300307. The patients/participants provided their written informed consent to participate in this study.

## Author Contributions

AS, RMA, MA, and RFA designed the idea of the conception, performed the methodology, formal analysis, data curation, and supervision besides revision of the manuscript. RB and EE participated in the methodology, formal analysis, data curation, and contributed their scientific advice. AS, RMA, and EE drafted the manuscript and prepared the manuscript for publication and revision. All authors have read and agreed to the published version of the manuscript.

## Funding

This work was supported by the Taif University Researchers Supporting Program (Project number: TURSP-2020/269), Taif University, Saudi Arabia.

## Conflict of Interest

The authors declare that the research was conducted in the absence of any commercial or financial relationships that could be construed as a potential conflict of interest.

## Publisher's Note

All claims expressed in this article are solely those of the authors and do not necessarily represent those of their affiliated organizations, or those of the publisher, the editors and the reviewers. Any product that may be evaluated in this article, or claim that may be made by its manufacturer, is not guaranteed or endorsed by the publisher.
